# Mortality from cancer in relation to smoking: 50 years observations on British doctors

**DOI:** 10.1038/sj.bjc.6602359

**Published:** 2005-01-25

**Authors:** R Doll, R Peto, J Boreham, I Sutherland

**Affiliations:** 1 Clinical Trial Service Unit & Epidemiological Studies Unit, Harkness Building, Radcliffe Infirmary, Oxford OX2 6HE, UK

**Keywords:** smoking, mortality, prostate, colorectal, alcohol

## Abstract

A total of 34 439 male British doctors, who reported their smoking habits in November 1951, were followed, with periodic up date of changes in their habits, until death, emigration, censoring. or November 2001. Information was obtained about their mortality from 28 of the 30 types of cancer in men reviewed by the International Agency for Research on Cancer (no death was recorded from the other two). In all, 11 of the 13 types in men that the Agency classed as liable to be caused by smoking were significantly related to smoking and the findings for the other two, which caused only few deaths, suggested they might be. Of the 13 types in men for which the Agency found only sparse or inconsistent data and for which we had data, only two appeared to be possibly related (one positively, one negatively), and the 638 deaths for the summed group were clearly unrelated to smoking. Of the two types for which the Agency thought that the relationship with smoking might be due to bias or confounding, the findings for one (prostate cancer) tended to support the belief that smoking was unrelated, and those for the other (colorectal cancer) showed a weak relationship with smoking, which (in a small subset) could not be attributed to confounding with the consumption of alcohol.

Knowledge of the effect of tobacco smoking in the production of cancer has increased progressively over 200 years until the International Agency for Research on Cancer (2004) was able to conclude that it contributed, to a greater or lesser extent, to the production of 15 types of the disease. Of the 19 other types that the Agency reviewed, two were commonly reported to be positively related to smoking, but the Agency thought that the positive findings might be attributable to bias in one case (prostate cancer) and to confounding in the other (colorectal cancer). For one type of cancer, the evidence showed a negative relationship with smoking, which indicated a protective effect (endometrial cancer), while for the other 16, the evidence either indicated no relationship at all (breast cancer in women) or was inconsistent or sparse (cancers of the salivary glands, small intestine, gallbladder and bile ducts, soft-tissue sarcoma, melanoma, other skin cancers, cancers of the ovary, testis, central nervous system, thyroid, and adrenal, Hodgkin's lymphoma, other lymphomas, lymphatic leukaemia, and myeloma).

In reaching these conclusions, the Agency included *inter alia* the published evidence for 14 types of cancer from observations on the mortality of 34 400 male British doctors over 40 years (Doll *et al*, 1994). Some additional evidence has, however, now been obtained by following the cohort for a further 10 years and we report here our observations relating to tobacco smoking over a 50-year period (1951–2001) for 28 of the 30 types of cancer that occur in men, which the Agency reviewed. During this period, information about smoking habits was updated periodically (in 1957, 1966, 1971, 1978, and 1991) with response rates varying between 94 and 98%. A more detailed account of the population and the methods of follow-up and analysis are described elsewhere (Doll *et al*, 2004).

## FINDINGS

### Cancers liable to be caused by smoking

Table 1 shows the mortality rates in relation to smoking habits of 13 types of cancer in men that the Agency classed as liable to be caused by smoking. A 14th (cancer of the ureter) was not classed separately in our data but included with cancer of the bladder. Table 1 also includes data for cancers of unspecified site, as many of them are likely to have been one or other of the major types that are commonly caused by smoking.

Inspection of the table shows that eight of the specified types of cancer and cancers of unspecified type were all clearly related to smoking, in that there were statistically significant positive trends in the mortality rates from lifelong nonsmokers through light and moderate cigarette smokers to heavy cigarette smokers and from nonsmokers through ex-smokers to continuing smokers. For one other type (myeloid leukaemia), there was a significant trend in the former but not in the latter, and for two others (stomach and kidney), there were significant trends in the latter but not in the former. The two remaining types (nasopharyngeal and nasal cancers) contributed only small numbers of cases (4 and 6, respectively), but the evidence, such as it is, suggests positive relationships.

### Cancers with indefinite relationships with smoking

Table 2 shows comparable data for the two cancers for which the Agency thought the relationship with smoking might be due to bias or confounding.

### Prostate cancer

The question of bias arose in the Agency's review because a positive relationship between smoking and prostate cancer was seen in many sets of mortality data but not in incidence data. Prostate cancers tend to occur at older ages than other cancers and often persist for many years before death. In these circumstances, it was thought that death that was actually due to (say) intercurrent pneumonia or vascular disease, both related to smoking, might be attributed on the death certificate to a prevalent prostate cancer, thus causing an artificial relationship with smoking that would not be seen in incidence data. Our data, now based on nearly 900 deaths, show some increasing risk with increasing numbers of cigarettes smoked per day, but only among smokers; there is no appreciable difference between nonsmokers and continuing smokers and the lowest risk is recorded in ex-smokers. They tend, therefore, to support the idea that smoking is unrelated to the disease.

### Large bowel cancer

Cancers of the large bowel have frequently been related to both smoking and the consumption of alcohol (Corrao *et al*, 2004; International Agency for Research on Cancer, 2004) and, as smokers tend to be heavier drinkers than nonsmokers, it seemed possible that the relationship observed with smoking might be an artefact due to confounding with alcohol. In our data, colorectal shows some relatively weak relationship with smoking with an implausibly strong relationship for rectal cancer and no evident relationship for colon cancer. The apparent limitation of the risk to rectal cancer may well, however, be largely or wholly due to an extreme effect of chance, as no such limitation was found in the many other studies reviewed by the International Agency for Research on Cancer (2004).

We have data for alcohol consumption for the last 23 years of the study (1978–2001) and for this period are able to show both data by smoking habits adjusted for drinking habits (none, occasional, 1–14 units a week, 15–28 units a week, and 29 or more units a week) and data by drinking habits, adjusted for smoking (in both cases the rates are adjusted for 5-year calendar periods; all other rates are adjusted for single year periods). With the reduced numbers available for the shorter period of observation (23 years), fewer of the differences observed are statistically significant. There continues to be some evidence that rectal, but not colon, cancer is related to smoking and this persists after adjusting for alcohol consumption, although the trend is no longer quite statistically significant. There is, however, no suggestion that it might be related to alcohol after adjusting for smoking (*χ*2 for trend, nondrinker, occasional drinker, regular drinker 0.56; for amount drunk by regular drinker, 0.95).

The reduction in the absolute number of deaths in the later period is greater than the number expected from the reduction in the number of years under observation partly because of the attenuation of the population by death with the passage of time and partly because the mortality from colorectal cancer in the UK was progressively reduced over the period of observation (WHO mortality database, 2004).

### Cancers unrelated to smoking

Table 3 shows data similar to those in Table 1 for the relationship with smoking of 13 of the 14 types of cancer in men that the Agency was unable to classify because the data were inconsistent or sparse and also for the group of 13 cancers as a whole. For the 14th type (adrenal cancer), we had no evidence as no death was attributed to it. For only three types are the numbers substantial (non-Hodgkin's lymphoma, cancer of the central nervous system, and myelomatosis) and for none of them is there the slightest suggestion that smoking either increases or decreases the risk of developing the disease. Nor is there any suggestion that smoking affects the incidence of any of the other types of cancer, for each of which we have only 50 deaths or fewer, except for Hodgkin's lymphoma and thyroid cancer. For the former, there is a weak suggestion that smoking might be protective (based on 26 deaths), while for the latter, there is a very weak suggestion that it might be causative (based on only eight deaths). When, however, so many tests are applied, some statistically significant results must be expected to occur by chance. What is more impressive is the lack of any suggestion of an effect of smoking on this group of cancers taken as a whole, based on the substantial total of 638 deaths.

## CONCLUSION

Data from following up a cohort of male British doctors relating smoking habits to the risk of 28 types of cancer are generally in accord with the conclusions of the recent review by the International Agency for Research on Cancer (2004) and help to clarify some of the relationships that were uncertain.

For two major types of cancer, the Agency was unable to reach a conclusion. For one (cancer of the prostate), our data suggest that it is unlikely to have any material relationship with smoking. For the other (colorectal cancer), our data confirm a relatively weak relationship with smoking, which could not be explained by confounding with the consumption of alcohol.

Data from 12 of the 13 types that occur in men that the Agency thought were caused by smoking support the conclusion, while those for 12 of the 13 types for which the Agency had sparse or inconsistent data suggest, when summed, that no special relationship existed. No data were available for the other two types of cancer in these two categories (respectively, cancer of the ureter and cancer of the adrenals).

## Figures and Tables

**Table 1:** Cause-specific mortality by smoking habit, standardised indirectly for age and study year: cancers related to smoking

		**Mortality per 100 000 per year**								**Standardised tests or trend (*χ*^2^ on 1 df)^a^**	
		**Lifelong nonsmoker**	**Cigarette smokers, no other habit**					**Other smokers**			
			**Former**	**Current**	**Current, cigarettes per day**			**Former**	**Current**	**N/X/S**	**Amount**
**Type of cancer**	**No. of deaths**				**1–14**	**15–24**	**⩾25**				
Oral	43	1.9	1.3	7.1	4.0	3.7	15.9	4.4	6.8	10.32	8.53
Oro- and hypopharyngeal	34	0.0	1.7	6.7	4.5	0.0	19.4	1.7	7.1	23.38	23.17
Nasopharynx	4	0.0	0.5	0.4	0.0	0.0	1.6	0.7	0.3	0.04	1.74
Nose and nasal sinuses	6	0.5	0.0	1.5	0.0	0.0	4.8	1.1	0.4	0.19	3.18
Larynx	40	0.0	2.6	10.3	6.0	8.5	17.3	2.9	4.7	17.46	25.38
Lung	1052	16.9	68.8	249.0	130.6	233.8	415.2	69.8	129.8	363.47	451.05
Oesophagus	207	5.7	20.1	34.4	21.2	34.4	50.0	18.9	25.1	29.40	37.49
Stomach	324	28.1	25.4	41.9	38.5	47.6	38.8	28.1	37.5	8.75	2.60
Pancreas	272	20.6	30.5	39.4	37.9	31.2	52.9	15.9	32.1	11.93	8.39
Liver	74	4.4	5.7	13.6	10.7	2.6	31.3	8.1	8.3	5.26	9.80
Kidney	140	9.3	13.2	16.2	16.4	16.6	15.5	12.1	18.2	6.92	1.61
Bladder	220	13.7	22.6	38.8	37.7	31.8	51.4	14.5	24.5	15.90	16.45
Myeloid leukaemia	100	6.3	12.6	11.3	2.8	14.0	18.3	8.8	10.6	1.98	7.47
Site unknown	269	19.1	21.8	44.2	29.9	61.0	38.2	20.1	38.7	27.62	13.68

a Values of *χ*^2^ on 1 df (degree of freedom) between lifelong nonsmokers, ex-smokers and continuing smokers and between lifelong nonsmokers and light, moderate and heavy cigarette smokers.

**Table 2:** Cause-specific mortality by smoking habit, standardised indirectly for age and study year: cancers uncertainly related to smoking

		**Mortality per 100 000 per year**								**Standardised tests for trend (*χ*^2^ on 1 df)^a^**	
		**Lifelong nonsmoker**	**Cigarette smokers, no other habit**					**Other smokers**			
			**Former**	**Current**	**Current, cigarettes per day**			**Former**	**Current**	**N/X/S**	**Amount**
**Type of cancer**	**No. of deaths**				**1–14**	**15–24**	**⩾25**				
Prostate^b^	878	89.4	80.9	90.0	66.7	99.6	113.3	85.9	94.9	0.67	0.52
Colorectal^b^	750	52.5	76.1	81.9	73.2	66.6	116.8	81.6	77.7	7.86	14.27
Colon^b^	549	41.1	58.6	54.5	57.2	46.6	62.3	63.2	52.2	0.89	1.60
Rectum^b^	201	11.3	17.5	27.1	16.3	19.9	53.5	18.3	25.4	14.91	25.99
Colorectal^c^	263	81.8	142.1	132.8	195.6	148.8	30.8	159.4	122.1	2.53	0.39
Colon^c^	202	61.4	117.3	82.4	120.5	105.3	0.0	126.4	80.1	0.47	0.00
Rectum^c^	61	20.4	24.9	51.2	77.1	43.9	30.5	33.1	42.3	4.26	1.38
Rectum^c^^,^^d^	61	21.4	24.9	46.8	72.3	39.8	27.5	33.7	40.4	3.46	0.01

a Values of *χ*^2^ on 1 df (degree of freedom) between lifelong nonsmokers, ex-smokers and continuing smokers and between lifelong nonsmokers and light, moderate and heavy cigarette smokers.

b 1951–2001.

c 1978–2001.

d Adjusted for alcohol.

**Table 3:** Cause-specific mortality by smoking habit, standardised indirectly for age and study year: cancers for which the evidence has been inconsistent or sparse

		**Mortality per 100 000 per year**								**Standardised tests for trend (*χ*^2^ on 1 df)^a^**	
		**Lifelong nonsmoker**	**Cigarette smokers, no other habit**					**Other smokers**					
			**Former**	**Current**	**Current, cigarettes per day**			**Former**	**Current**	**N/X/S**	**Amount**		
**Type of cancer**	**No. of deaths**				**1–14**	**15–24**	**⩾25**						
Salivary glands	12	1.2	0.4	0.0	0.0	0.0	0.0	0.9	2.6	1.06	1.42
Small intestine	20	2.1	1.3	3.2	3.9	3.3	2.3	3.6	0.7	0.36	0.01
Gallbladder	31	2.8	3.1	3.5	10.0	0.0	0.0	3.2	2.9	0.03	1.98
Soft-tissue sarcoma	20	3.7	0.9	2.4	6.3	0.0	0.0	2.2	1.6	1.25	2.50
Melanoma skin	44	3.8	2.8	6.2	6.4	7.6	3.8	6.0	4.1	0.22	0.63
Nonmelanoma skin	17	2.0	0.8	0.8	0.0	2.3	0.0	2.1	2.4	0.01	0.06
Testis	12	0.5	1.5	1.4	2.7	0.0	1.9	2.1	0.8	0.04	0.96
Central nervous system	125	12.6	15.9	9.7	7.5	8.2	14.1	11.8	11.9	0.67	0.10
Thyroid	8	0.0	1.3	0.7	0.0	0.0	2.7	0.6	1.0	0.98	4.44
Hodgkin's lymphoma	26	4.4	2.9	0.5	1.4	0.0	0.0	1.4	3.6	2.02^b^	7.29^b^
Non-Hodgkin's lymphoma	175	15.9	15.8	21.1	23.1	29.1	7.0	17.5	18.7	1.13	0.00
Myelomatosis	98	11.3	10.5	10.2	4.9	14.0	10.8	9.9	7.8	1.00	0.04
Lymphatic leukaemia	50	5.8	4.5	6.3	0.0	12.5	4.9	4.2	5.6	0.04	0.26
All above	638	65.9	61.0	62.6	69.2	66.0	49.7	65.5	63.5	0.10	0.62

a Values of *χ*^2^ on 1 df (degree of freedom) between lifelong nonsmokers, ex-smokers and continuing smokers and between lifelong nonsmokers and light, moderate and heavy cigarette smokers.

b In direction of prevention.
